# Useful distracting information: ERP correlates of distractors in stimulus-response-episodes

**DOI:** 10.1371/journal.pone.0206468

**Published:** 2018-11-01

**Authors:** Lea Donata Priester, Daniel Wiswede

**Affiliations:** 1 Department of Neurology, University of Lübeck, Ratzeburger Allee, Lübeck, Germany; 2 Department of Ophthalmology, University Medical Center, Johannes Gutenberg-University, Mainz, Germany; National University of Ireland Maynooth, IRELAND

## Abstract

A basic process in regulating behavior that helps us to disentangle meaningful from distracting information is the binding of stimulus and response features into stimulus-response episodes or “event files”. Recent studies have shown that even irrelevant information is bound into event files; distractor repetition on the next trial can trigger the response encoded in this episode, which is indicated by faster reaction times. The present study was conducted to get further insight into the electrophysiological underpinnings of those distractor-based retrieval. For that, we analyzed the N2, a negative deflection in event-related potentials that has been associated with a multitude of processes occurring when relevant and irrelevant stimuli compete with each other within a given trial or even in sequences of trials. Our study showed that distractor which did not provide useful information regarding the required behavior led to more negative N2 amplitudes, whereas distractors that provide useful response-related information were associated with less negative N2 amplitudes. Our results are explained as an adaptive mechanism that helps to hedge against invalid stimulus-response-bindings before an error occurs to increase efficiency of human behavior.

## Introduction

We know from every day experience that not only task-relevant, but also distracting information influences our performance. It is processed to some extent; sometimes it yields additional information which might be useful for goal-achievement. The exact nature of brain processes evolved to deal with important task-relevant details in an overwhelming magnitude of distraction is still highly debated. This paper aims to bring further insight into the behavioral and neurophysiological underpinnings of task-irrelevant information that sometimes, but not always is useful for the current task.

Among the most often used tasks to examine processing of relevant in the presence of irrelevant information are variations of the classical Flanker task [1]: target stimuli are mapped to specific responses while distracting stimulus components call either for the same (= congruent trials) or different (= incongruent trials) responses. A typical finding on the single- trial level is that participants respond more slowly when distracting information is associated with an alternative response. This “congruency effect” is normally explained by response conflict caused by automatic co-activation of the response channel associated with the target and with the distractor, leading to slower correct responses and a higher error rate for congruent trials. Thus, it is a strong behavioral indicator that also task-irrelevant information on the single-trial level are processed to the point “where they are identified enough to tend to elicit appropriate responses” [1].

An intensively examined neurophysiological marker of this response conflict within a single trial is the N2, a negative frontocentral deflection in event-related potentials (ERPs) starting around 200 ms after stimulus onset. Very consistently among many studies, the N2 has been shown to be larger (more negative) in amplitudes for incongruent relative to congruent trials [2, 3].

Goal-directed behavior requires flexible responding to a stream of upcoming information. Previous stimuli should therefore be considered while preparing to respond to upcoming stimuli. In line with this, a large amount of research has focused on how previous (= prime) trial congruency influences current (= probe) trial processing [4–6]. The critical question is whether a conflict emerging at a prime trial influences cognitive control processes on subsequent probe trials. In ERPs, the N2 appears to be also sensitive to previous trial congruency. According to the conflict conflict-control loop theory or conflict monitoring hypothesis [7], the N2 should be less pronounced for high conflict probes preceded by high conflict primes, suggesting decreased conflict activation; a result that is sometimes [8], but not always found when controlling for identical stimulus-response- repetitions [3]. Taken together, the general pattern how prime trials influence N2 amplitude in the probe remains controversial and less clear and might be influenced by multiple processes related to stimulus- and response- repetitions [3, 8–10], changes in attention triggered by congruent information [10], conflict frequency [11], negative priming [12, 13], and probably others.

The current study was conducted to bring further insight into the factors that are crucial for sequential N2 modulation. We think that also retrieval of previous stimulus episodes elicited by distractor repetition might contribute to N2 amplitude change. This idea is derived from a group of behavioral studies which discussed distractors as being not only conflict generators, but simultaneously being useful information providers. Previous research used an experimental design that allows to disentangle distractor inhibition effects from distractor based response retrieval [14]. The basic idea is grounded on binding theories, which provide evidence that stimulus and response are bound together [15–17] in a single event file in episodic memory. Upon next encounter of the stimuli, the event file is automatically retrieved from memory, leading to faster and more efficient responses. Extending those binding theories, it was shown that also irrelevant distracting information presented simultaneously with the target can be bound with the response into the event file and influences next stimulus processing [14, 18]. Thus; although the distractor competes with the target stimulus on the one hand, it can become integrated with target response into an event file to provide useful information on next encounter and facilitate retrieval of the prime response. This distractor based response retrieval is indicated by significant interaction of response relation (response repetition vs. response change) and distractor relation (distractor repetition vs. distractor change), with the advantage of distractor repetition being larger in response repetition relative to response change trials. Thus, distractor repetition leads to an advantage in response repetition trials and to a disadvantage (or smaller advantage) in response change trials [14, 18]. In addition to distractor based response retrieval, inhibition is indicated by an general advantage (significant main effect) of distractor repetition, since repeating an stimulus that was already inhibited in the prime is beneficial for reaction times [18].

Integrating irrelevant information into event files can be seen as an adaptive mechanism of the cognitive system which allows redundancy gains and implicit learning: Irrelevant stimulus features are sometimes informative since they co-occur with relevant features. However, distractors are called distractors because they are far from being perfect predictors for appropriate behavior, making distractor-response-bindings error-prone. Wiswede and co-authors [19] examined the neuropsychological mechanisms associated with erroneous distractor-based response- retrieval. The study indicated that the error-related negativity (ERN), an ERP index of error processing [20, 21], is enhanced for errors resulting from a distractor-based retrieval of inadequate previous responses. It was concluded that this ERN enhancement indicates stronger cognitive control process, which allows to detect and possibly correct the source of this error after an error occurred.

The current study was conducted to foster our understanding of processes that help to retrieve useful distractor-response- bindings from an event file and, alternatively, identify no longer useful distractor-response bindings. For this, the study of Wiswede and colleagues [19] was replicated with a new sample of participants. Again, a sequential priming paradigm based on a modified flanker-task was used and possible confounds based on known sequence effects were carefully controlled by restrictions in target-distractor-sequence selection (see methods). A complex 8-letter-to four button matching was used, since previous research with 4-letters to 2 button matching does not allow to separate useful distractor repetitions (in which the probe distractor helps to retrieve the probe target) from useless distractor repetitions (in which the probe distractor retrieves conflicting information from the probe target) from other important mechanisms influencing the N2 amplitude (i.e. trial congruency, negative priming, conflict adaptation). In contrast to the previous study using the same experimental design [19], the current study aimed to examine sequences of correct rather than erroneous response retrieval. To achieve longer sequences of correct responses not interrupted by errors, participants speed-accuracy trade-off was shifted toward slower responses and lower error rates by providing a learning procedure prior to experimental measure.

For reaction times, we expect to replicate earlier findings [14] showing distractor-response-binding. In detail, we assume that distractor repetition is useful in response repetition trials and less useful or detrimental in response change trials, because the distractor repetition helps to retrieve a compatible response. Additional distractor inhibition effects should be indicated by an additional general response time advantage for distractor repetition relative to distractor change. Analog to expected behavioral results, we assume that distractor-response binding should also affect ERP amplitudes. Namely, the N2 could be sensitive to indicate whether distractor-response-bindings established in the prime are still valid in the probe. If the same response is required in the prime and in the probe, increased performance due to distractor repetition should diminish N2 amplitude relative to distractor change, because the distractor retrieves the correct response and there is no need to change or update the previously established distractor-response-binding. To make sure that the predicted N2 decrease for distractor repetition is not caused by target identity repetition, an 8-targets to 4- buttons- matching was used that allows to analyze response-repetition trials without repetition of the same target identity. In other words, there is a target identity change in response repetition as well as in response change probes. In both response condition, the distractor either repeats or changes from prime to probe. In response repetition trials, distractor repetition decreases reaction times and N2 amplitude because the distractor retrieves the appropriate response. In response change trials, distractor repetition does not provide helpful information for response generation or suggests even an erroneous response, indicated in increased reaction times and increased N2 amplitudes, indicating the need to update the DR- binding from prime to probe.

## Materials and methods

### Participants

Data are reported from 21 participants (10 female, age range 21–30; data were originally recorded from 25 participants, four of them were excluded from further analysis, two because there were too few trials in one of the relevant categories for a reliable N2 analysis (less than 20, see [22]), two because of uncorrectable artifacts.) To ensure high performance level needed for low error rates, all participants were students of medicine and therefore experienced with long-lasting demanding tasks. All participants had normal or corrected to normal vision. The experiment was approved by the ethics committee of the University of Lübeck. All participants provided written informed consent prior to the investigation and were reimbursed with 8 €/h (average 16 €) after completion of the experiment. The study conforms with The Code of Ethics of the World Medical Association (Declaration of Helsinki), printed in the British Medical Journal (18 July 1964).

### Stimuli and procedure

**Fig 1:**
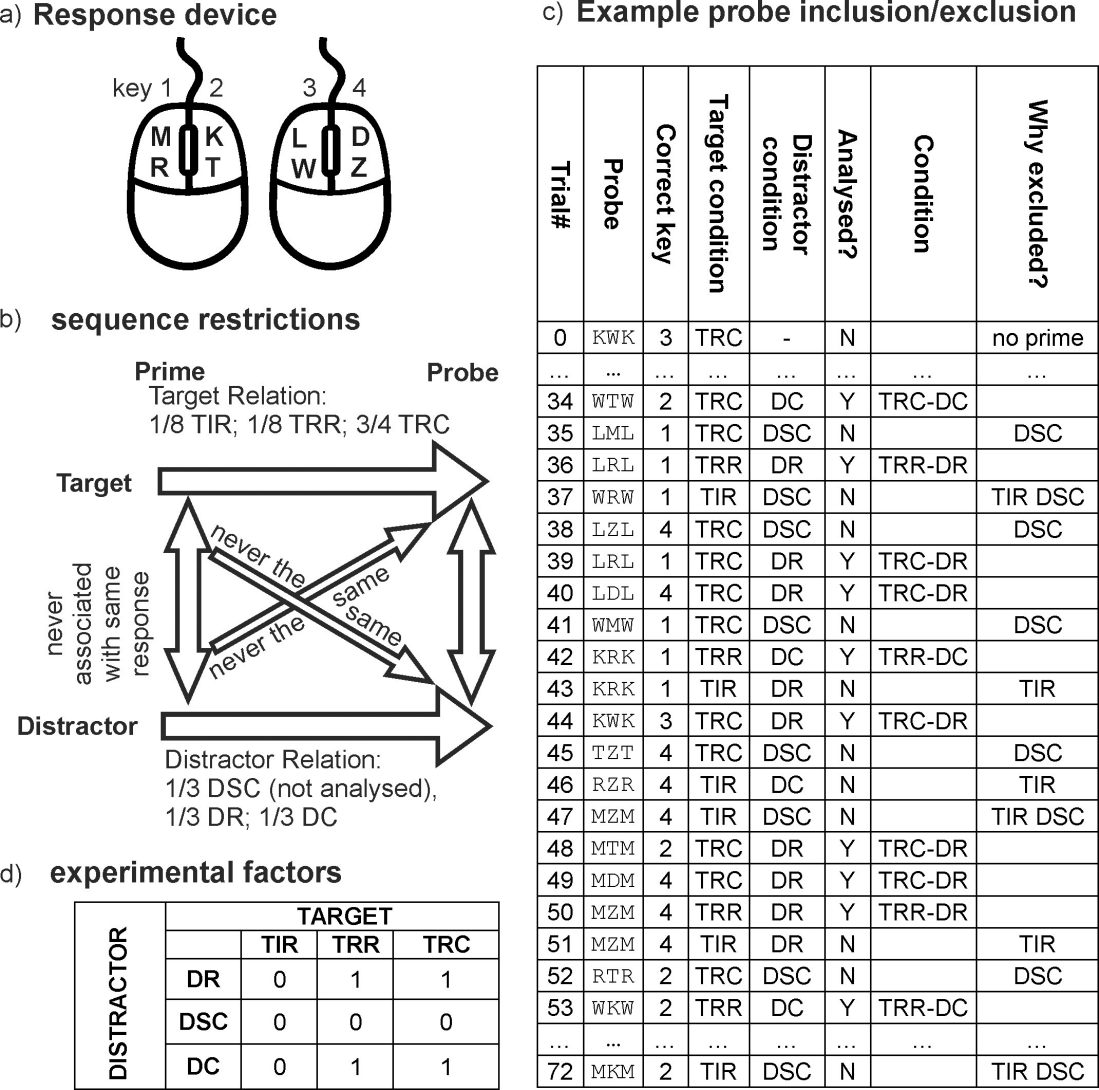
Description of the flanker task. (A) response device with 8-letters to 4-button-matching. (B) sequence restrictions. prime-probe-sequences regarding target-distractor-relation and prime-probe-relation. Target relation: TIR = Target Identity Repetition (identical target in prime and probe), TRR = Target Response Repetition (prime and probe target were different in identity, but were mapped on the same response button), TRC: Target Response Change (prime and probe target were associated with different responses). Distractor relation: DSC = Distractor Same Category (distractor in prime and probe were different, but associated with the same response), DR = Distractor Repetition from prime to probe; DC = Distractor Change (distractors in prime and probe were associated with different responses). See text for detailed explanation of sequence restrictions. (C) a fraction of a possible block, indicating included and excluded trials. abbreviations same as in b. Y = Yes, N = No. (D) experimental factors. 0 = excluded from analysis, 1 = analysed. abbreviations as in b.

Participants conducted a flanker categorization task with eight letters assigned to four buttons approximately 90 cm from a 17 inch CRT monitor. The experiment was a new implementation of the Wiswede et al., [19] experiment, now programmed in PsychoPy in Python programming language [23]. Each flanker stimulus comprised of a central target letter surrounded by three identical distractor letters on each side. Targets and distractors comprised eight consonants (M, R, K, T, D, Z, L, W). Participants were instructed to respond as fast and as accurate as possible to the target stimuli by pressing one of four response buttons of on two computer mice. Although the response device was different to that in Wiswede et. al., [19], the hand-and finger-mapping was the same: two consonants were always mapped to one response button (MR = left middle finger or key 1, KT = left index finger or key 2, DZ = right middle finger or key 3, LW = right index finger or key 4; see Fig 1A). Each trial started with presentation of a flanker stimulus for 150 ms, followed by a blank screen for a maximum of 750 ms. If the response did not occur within the 900 ms after stimulus onset, the next trial was presented. The experiment consisted of 16 blocks with 73 trials each, interrupted by a short break. Since we were interested in effects caused by previous trial properties, every trial served as a probe (trial of interest for the analysis) as well as a prime (trial preceding the trial of interest). The first trial of each block was removed from further analysis, because it did not have a direct preceding stimulus. Thus, each participant was presented with 16 x 72 = 1152 prime-probe-pairs.

### Control for known sequential effects

The continuous succession of primes and probes required to control for several sequence effects known to influence response times and accuracy in sequential priming tasks. First, we avoided target-distractor-compatibility since response-congruency between targets and distractors has a major impact on response times and accuracy in the current and subsequent trials and influences N2 amplitude [2] beyond the scope of the current research question. Thus, all flanker stimuli were response incongruent; that is target and distractor stimuli were never the same and were never assigned to the same response button. This also controls for conflict adaptation effects [3, 8], since prime and probe trials should therefore not differ in conflict level.

Second, we balanced prime-probe target relations. One-fourth of the probe responses required a prime response repetition (1/8 = response repetition with same target (= TIR; Target Identity Repetition; excluded from analysis, see below), 1/8 repetition of the other target letter that was mapped to the same response button (= TRR; Target Response Repetition without target identity repetition)), 3/4 of the probe responses did not repeat the prime response (= TRC; Target response change). Third, we balanced prime-probe distractor relation orthogonally to target relation. In 1/3 of all trials, the probe distractor was an exact repetition of the prime distractor (= DR; Distractor Repetition), in 1/3 of the trials the probe distractor was different, but associated with the same response as the prime distractor (= DSC; Distractor Same Category, excluded from further analysis, see data analysis section), in the remaining 1/3 of the trials, there was no response-association between prime and probe distractors (= DC; Distractor Change). Fourth, we avoided distractor-to-target repetitions because repetition of the prime distractor as the probe target typically leads to slowed responses (negative priming); the same holds true for target-to-distractor repetitions. Fifth, we balanced distribution of response buttons across both hands and index/middle fingers. As mentioned above, targets and distractors within a trial were never mapped on the same response, but in 1/3 of all trials, the distractor was associated to a response that belonged to the same hand (different finger), to the different hand (same finger), or to the different hand (different finger) as the target, respectively. This factor is not of theoretical interest and will not be further analyzed. Beyond those restrictions, selection of flanker stimuli was random so that the prime trial did not allow a above chance prediction regarding the probe properties. For an illustration of the rules and constraints of sequence construction, see Fig 1B.

To meet all those experimental requirements, the same 1000 blocks with 73 trials each as in Wiswede et. al., [19] were used. They were generated using a special purpose Java based program. Within each block, all types of trial sequences occurred in a randomized order. Out of this pool, 16 blocks were drawn randomly (without replacement) for each participant and served as the basis for stimulus presentation.

### Instruction and practice

Participants received a written explanation of the experimental task and were asked to remember the buttons associated with the eight letters. After they were able to recall the button presses correctly, participants worked through a series of practice procedures prior to the experiment. First, participants learned the letter-to-button-matching by reacting to single letters from the response set presented on the screen. The letter was shown until correct button press and replaced by a new letter from the set. Errors resulted in the message “wrong key”. This procedure was continued until there was a sequence of 100 correct responses. Second, a series of response device pictures was presented on the screen with one of the buttons on each picture was marked by color. Participants were required to name the two letters associated with the key. The experimenter continued with the next picture until there were 16 correct answers given. Third, the same flanker stimuli with target and distractors as in the experiment were presented, but without time pressure. Participants forwarded to the next flanker trial by correct button press, erroneous responses were accompanied by an error message and the chance for a new try. The fourth practice session was very similar to the experiment except that a visual feedback screen was provided for erroneous responses (“error!”) and for slow responses (RT > 900 ms, screen “too slow”). After at least 5 minutes of practice, the last practice trial aborted if there was a minute with less than 10% slow or erroneous responses. The intensive practice procedures were conducted to make sure that participants produce less errors than in Wiswede (2013) to enable an analysis of sequences of correct responses not interrupted by errors. After that, participants were informed that feedback will not be provided during the experimental blocks; a short break until button press was provided, the EEG recording was started and the main experiment was started.

### Data recording

During the experiment, the electroencephalogram (EEG) was recorded from 29 electrodes including all 19 standard locations of the 10–20 system [24] with Ag-AgCl electrodes mounted in an elastic cap (Electro Caps International, Eaton, OH) relative to two reference electrode placed on both ear lobes. Eye-movements were recorded with electrodes affixed at the right and left external canthi (horizontal electrooculogram (hEOG), bipolar recording) and at the left and right orbital ridges (vertical electrooculogram (vEOG), bipolar recording). Impedances of all electrodes were kept below 5 kΩ. Biosignals were amplified with a band-pass from 0.05 to 30 Hz with a digitization rate of 250 Hz using Neuroscan amplifiers and Acquire recording software (Neuroscan Inc., Sterling, VA).

*Artifact correction and averaging*. Prior to ERP data analysis, all trials containing eye-movement artifacts were corrected using ICA-based artifact correction implemented in EEGlab [25, 26]. Stimulus-locked ERPs (time-locked to the onset of the flanker stimulus) were averaged for epochs of 1200 ms starting 200 ms prior to stimulus. A pre-stimulus period of 100 ms served as a baseline for ERP-computation. All ERP figures and all ERP statistics are slightly band-pass-filtered (0.1 to 30 Hz).

#### Data analysis

*Probe selection for analysis*. Behavioral and ERP analysis was restricted to a subset of prime-probe-sequences. The following probes were excluded from analysis:

- the first trial of each block, because there was no prime trial
- all trials with identical target repetitions from prime to probe (TIR-probes; = Target Identity Repetition) to exclude target identity repetition effects [18].
- all trials where prime and probe distractor were different, but associated with the same response (DSC-Trials, = Distractor Same Category), because it is unclear if distractor-associated response repetition without distractor-repetition impacts behavioral results. In addition, this procedure results in the same amount of trials included in distractor repetition and distractor change conditions.
- trials that were presented 6 seconds prior or three seconds after an error were excluded from ERP and behavior data analysis, because there are a couple of separate mechanisms occurring around error commission (i.e. pre-error speeding and post-error-slowing), which were not in the scope of this study. A possible sequence of correct trials with inclusion/exclusion criteria is depicted in Fig 1C.

*Experimental factors*: ERP and behavior data were submitted to a repeated-measurement-ANOVA. Based on prime target and distractor properties, the following two within subject factors were analyzed: TARGET (levels TRR (= Response Repetition) and TRC (= Response change), notice that target identity changed in both, TRR and TRC trials); DISTRACTOR (levels DR (= Distractor repetition) and DC (= Distractor Change)). See Fig 1D. For ERP data, we analyzed the mean amplitudes in a time window from 250–350 ms after stimulus onset (boundaries were determined by visual inspection and are in accordance with previous literature[2]). Statistical analysis was limited to two midline channels (factor ELECTRODE, levels Fz and Cz, same as in [3]) as visual inspection of the data showed that the effects of the experimental manipulations were most pronounced at these positions. Correction for non-sphericity with the Greenhouse-Geisser epsilon coefficient was performed whenever applicable. The reported p-values are corrected, effect size is provided as “partial eta squared” (_p_η^2^) and “generalized eta squared” [27] in the supporting information. ERP data were generated using ERPlab [28] and exported to RStudio [29, 30], all calculations were conducted with the R package ez [31], all figures depicting results were generated with ggplot2 [32].

## Results

### Behavioral data

**Fig 2:**
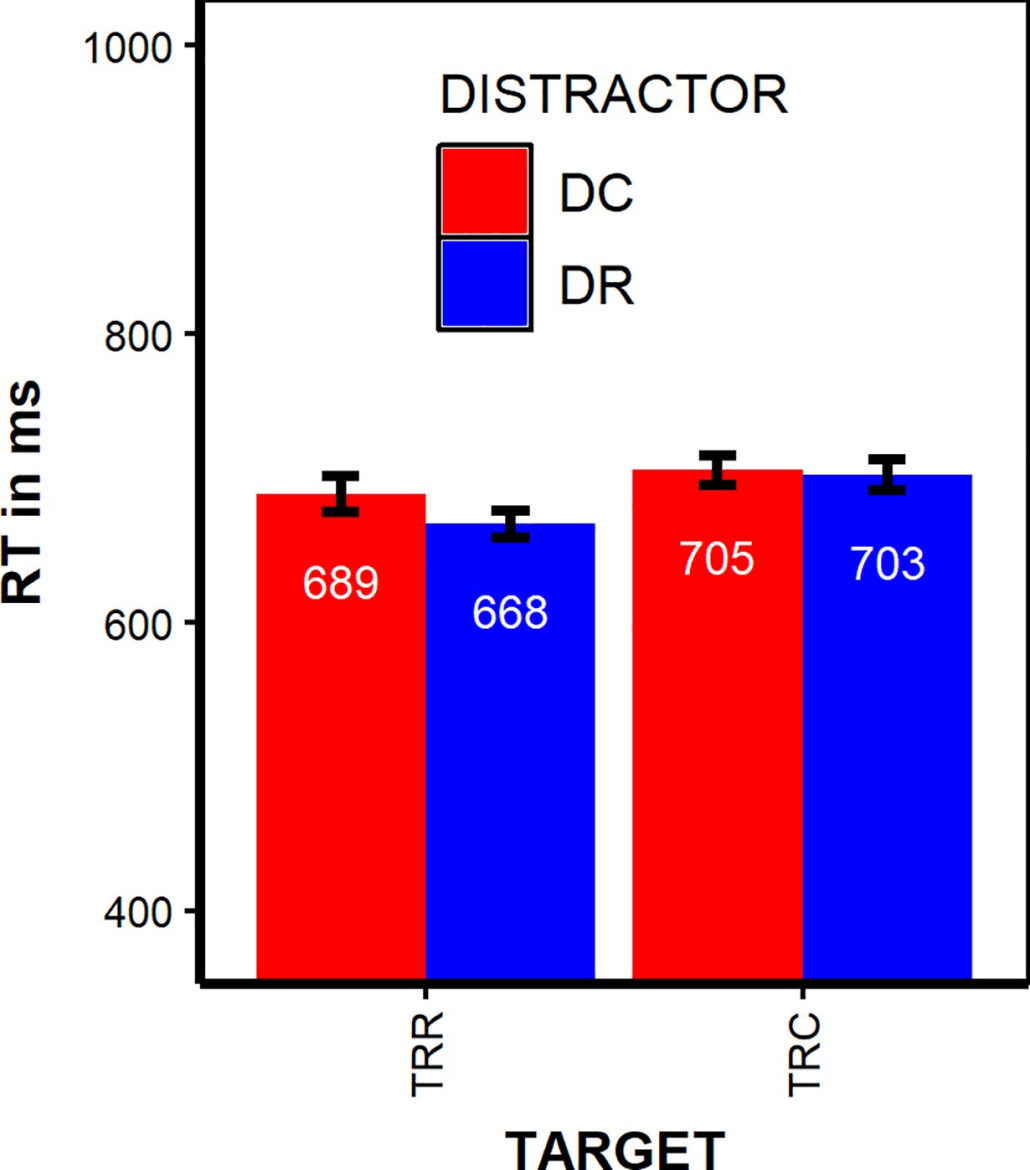
Reaction times. RTs separated by TARGET (TRR = Target Response Repetition, TRC = Target Response Change) and DISTRACTOR (DC = Distractor Change, DR = Distractor Repetition). Error bars indicate +/- 1 SE.

*Response retrieval effects in RTs*. Average RTs and error frequencies for all combinations of our factorial design are shown in Supplement S3 File or Table D in S1 File, reaction times are plotted in Fig 2.

In a 2 (TARGET) x 2 (DISTRACTOR) ANOVA with correct probe RTs as DV, both main effects were significant. Participants responded faster if the response repeated from prime to probe (679 ms) compared to response change trials (704 ms), *F*(1,20) = 10.53, *p* < .01, _p_η^2^ = 0.35. Distractor inhibition [18] is indicated by faster responses if the distractor repeated from prime to probe (685 ms) compared to distractor change probes (697 ms), *F*(1,20) = 8.09, *p* < .01, _p_η^2^ = .29. More importantly, there was a significant TARGET x DISTRACTOR interaction, *F*(1,20) = 7.24, *p* < .014, _p_η^2^ = .27. see Supplement S3 File or Table D in S1 File and Fig 2, showing that distractor repetition led to faster responses only for response repetition trials (in which the correct response was retrieved) but not for response change trials; that is, we observed the typical effect pattern that is indicative of distractor-response binding and retrieval. Since the training procedure was successful in reducing the amount of errors, we did not analyze error rates in details. See S3 File or Table D in S1 File for error rates in percent. See also Supplement S1 File to S3 File and S1 Fig for a more detailed analysis and for the data.

### ERP data

**Fig 3:**
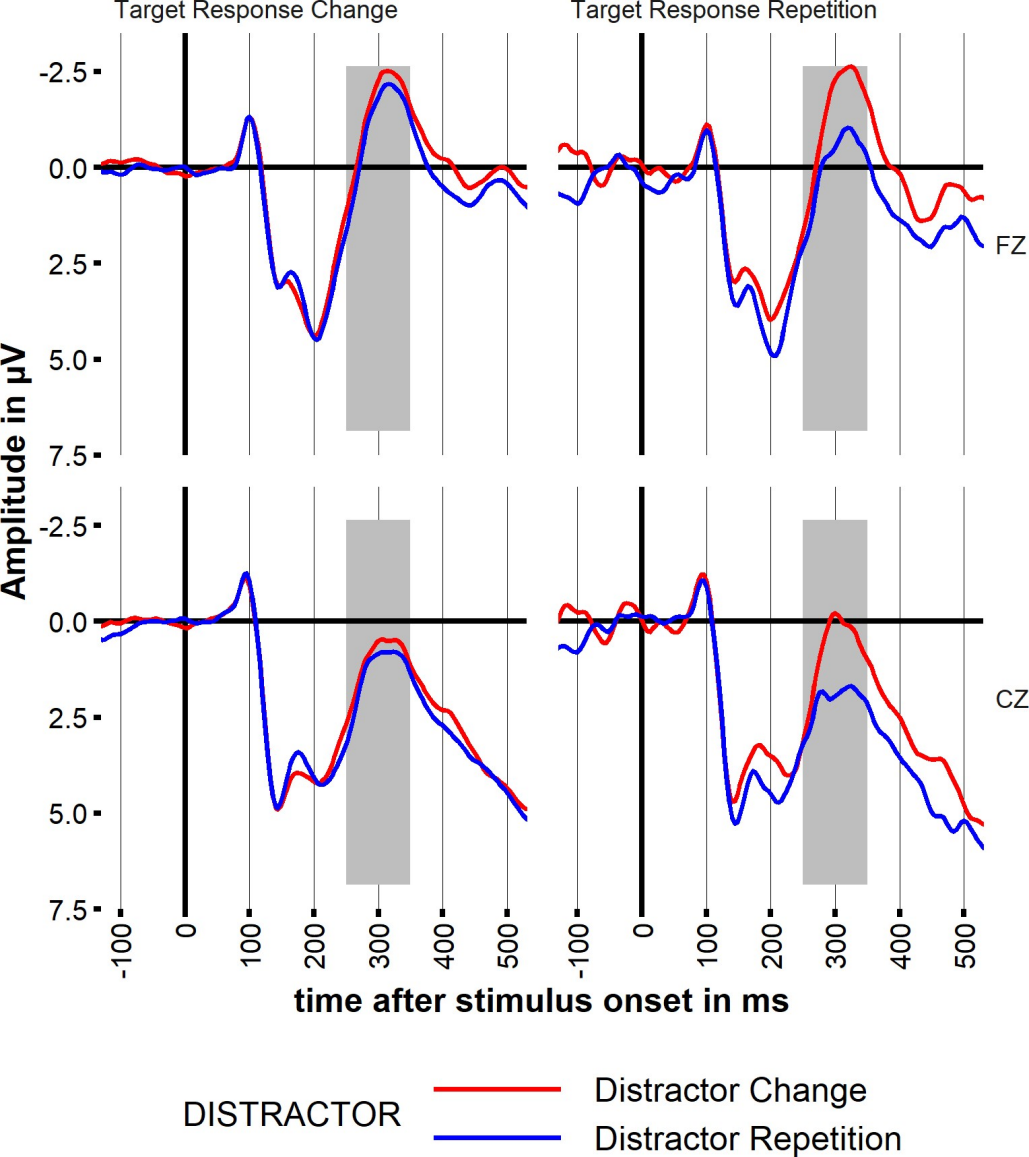
ERPs. Stimulus-locked event-related brain potentials (ERPs) on midline electrodes FZ and CZ separated by TARGET (Target Response Repetition (prime and probe target were different in identity, but were mapped on the same response button), Target Response Change (prime and probe target were associated with different responses). Lines represent DISTRACTOR (Distractor Repetition from prime to probe; Distractor Change (distractors in prime and probe were associated with different responses)). Baseline: 100 ms pre-stimulus. Shaded areas indicate time window for statistical analysis.

ERP data on FZ and CZ are shown in Fig 3 (see S2 Fig for all electrodes). Visual inspection of the data indicated a negativity within the N2 time window of interest (250–350 ms) which was strongest on midline frontocentral electrodes around Cz and Fz. Statistical analysis confirmed visual impression. Distractor inhibition effects are indicated in a general decrease in N2 amplitude for distractor repetition trials (main effect DISTRACTOR; F(1,20) = 13.80; p < .01, , _p_η^2^ = .41, mean amplitude 250–350 ms on FZ; DR: -0.6μV; DC: -1.5μV). The most critical ERP result for our hypothesis is a diminished negativity for response repetition probes with distractor repetition, seen on all displayed midline electrodes (interaction TARGET x DISTRACTOR: F(1,20) = 4.77; p < .04, _p_η^2^ = .19; post hoc comparisons based on two-way-interaction TARGET x DISTRACTOR showed large distractor effects for Target Response Repetition, but not for Target Response Change trials; t = 4.17, p_(Bonferroni)_ < .01; see Table P in Supplement S1 File for all post hocs and Table K in Supplement S1 File for descriptive statistics). Thus, if the distractor is beneficial in retrieving the previous response, this is indicated in a smaller N2 amplitude. See also S1 File and S4 File, S5 File, S2 Fig and S3 Fig for a more detailed analysis and for the data.

## Discussion

The present study applied a sequential priming paradigm to get further insight into the behavioral and electrophysiological effects of distractor-based retrieval. Error rates and reaction times indicate that our attempt to shift speed-accuracy-trade-off towards more correct responses was working; error rates were substantially lower and reaction times were higher than in our previous study [19]. On the behavior level, we replicated earlier results of distractor-based response retrieval, indicated by distractor repetition benefits in response repetition, but not in response change trials. Thus, irrelevant stimuli become associated with responses and retrieve these response episodes on a later occasion [14, 19]. However, the central question was to examine whether there are modulations of the well-known N2-component that could be associated with those distractor-based retrieval processes. More specifically, after previous research has shown that there are mechanism to detect and eliminate inadequate distractor-response- bindings after errors [19], we were interested in mechanism that help to distinguish useful from no longer required distractor-response-bindings in correct response sequences. In fact, our ERP results indicated that useful distracting information that is compatible with task-related behavior is associated with the smallest N2, whereas distractors that carry no longer valid information regarding the required response are associated with larger N2s. Behavior data and N2 amplitudes are astonishing similar: Distractor repetition decreases reaction times and N2 amplitudes. Fastest reaction times and smallest N2 amplitudes are associated with probes in which the distractor helps to retrieve the prime response, indicated by a significant interaction of TARGET x DISTRACTOR in behavior and N2 mean amplitude data.

Our behavior effects are easily explained by existing models. Decreased reaction times for response repetitions relative to response changes (main effect TARGET) even in the absence of stimulus identity repetition is an established finding [33] and has been intensively discussed earlier [34, 35]. It is also well-known that distractor repetition (main effect DISTRACTOR) is beneficial for reaction time; it can be explained by an inhibition account [18, 36]. The interaction of target relation and distractor relation replicates earlier findings on distractor-based response retrieval. For distractor repetition trials, the model assumes that two processes compete during probe processing: The algorithmic process of programming the probe response based on the processing of the target competes with an automatic retrieval process elicited by the probe distractor [19, 37, 38]. In distractor repetition probes that require response repetition, both processes point to the same behavior, indicated in reduced processing effort. In contrast, all other combinations of target and distractor properties analyzed here (distractor repetition with response change and both categories of response change trials) result in contrasting outcomes of the two processes. Overcoming this interference from incompatible information is indicated in longer processing times.

Our ERP results show that probes in which the distractor does not provide useful information regarding the required behavior are indicated by more negative N2 amplitudes, whereas distractors that provide useful response-related information (response repetition with distractor repetition) are associated with less negative N2s. We suggest that the N2 increase might reflect a mechanism for generating new distractor-response bindings, whereas N2 decrease indicates that there is no need to update existing memory traces. However, sometimes, the respond suggested by the previous distractor might be even so potent that the distractor-based response is given erroneously. As previously shown [19], those so-called retrieval errors goes along with an increased in the error related negativity, which, in this case, might reflect an retroactive mechanism to identify and neutralize distractor-response-associations that are no longer needed.

A main effort of the present study was to carefully control for alternative explanations for N2 changes, since the N2 amplitude has been shown to be sensitive to a multitude of within- and between- trial variations in sequential priming tasks [2, 3, 8–12]. In detail, our N2 cannot be explained by well-known congruency effects [2] or degree of conflict present on a given stimulus [8, 39], since target and distractor were always different in identity and never associated with the same response. In addition, previous research [11] has shown that a high frequency of incongruence trials is detrimental to N2 amplitude, indicating that within trial conflict cannot account for our findings. N2 amplitude changes can also not be influenced by target identity repetition, because we used an eight-letter-to four-button-matching task to separate target identity repetition from response repetition and excluded probes with complete stimulus repetition and with target identity repetition. Negative priming [40] can also not account for our N2 differences, since the current experiment did not include any probe targets that have been distractors in the prime. A classical explanation based on the conflict monitoring theory [3, 7, 8] can also not account for our N2 findings, because all of our primes and probes were incongruent and should therefore not differ in the level of within trial conflict. High conflict in the prime trial can therefore not increase attentional control to facilitate probe trial processing.

However, visual inspection indicates that our N2 looks very similar to previous N2 studies reporting that N2 amplitude is attenuated for probes that follow high conflict primes relative to low conflict primes [3, 8]. Therefore, it is conceivable that another kind of conflict, which emerges between target response selection and automatic retrieval process elicited by the probe distractor [19, 37, 38]. Because previous studies [3, 8] did not consider distractor repetitions or changes as separate categories, distractor-based response retrieval could contribute to controversies in previous studies.

Taken together, our data show that the cognitive system uses task-irrelevant redundancies in the selection of appropriate responses. A lot of previous research has shown that binding and retrieval in perception and action is a general phenomenon [15]. Integrating additional information into event files can be seen as the default configuration of the cognitive system because it allows for redundancy gains and implicit learning since even irrelevant features often correlate with relevant features due to their co-occurrence within certain objects. Given this fairly non-selective operation of memory and retrieval processes, it is crucial that the cognitive system has mechanisms to detect and eliminate inadequate stimulus-response episodes from memory. After we have shown that there are retroactive mechanism to correct for inadequate stimulus-response episodes after errors [19], the current study provided electrophysiological evidence for mechanisms to hedge against invalid stimulus-response bindings before an error occurs. Among other mechanisms, this might contribute to increased efficiency and flexibility in human behavior regulation.

## Supporting information

S1 File R Markdown output. provides more detailed information on all calculations.
(PDF)

S2 File Behavior data single subjects. Behavior data for all subjects and experimental conditions.
(TXT)

S3 File Behavior data group. behavior data on the group level. Reaction times and error rates in percent with SE and SE.
(TXT)

S4 File ERP data single subjects. ERP data on the group level. Reaction times and error rates in percent with SE and SE.
(TXT)

S5 File ERP data mean amplitudes. ERP data, mean amplitudes, time window 250–350 ms, midline electrodes, for all subjects.
(TXT)

S1 Fig Error rate in percent. (PDF)

S2 Fig ERPs on all electrodes. ERPs all electrodes by TARGET and DISTRACTOR.
(PDF)

S3 Fig N2 amplitudes. N2 mean amplitudes on midline electrodes FZ and CZ.
(PDF)
