# Supplementary material for: Useful distracting information: ERP correlates of distractors in stimulus-response-episodes
Source: PLoS One. 2018 Nov 1;13(11):e0206468. doi: 10.1371/journal.pone.0206468 (PMC6211706; doi:10.1371/journal.pone.0206468)
Supplement: S1 File — provides more detailed information on all calculations. (PDF) [file pone.0206468.s001.pdf]

Supporting information

Paper: Useful distracting information: ERP correlates of distractors in stimulus-response-episodes

Author: Lea Priester and Daniel Wiswede
Affiliation: University of Lübeck

submitted to PlosOne

02 Oktober, 2018

Contents

1 Demograpics **2**

2 Behavior data. **3**
2.1 behavioral data, loading and exporting. 3
2.2 Reaction times 4
2.2.1 Descriptives, reaction time and errors. 4
2.2.2 Bar graph reaction times. 5
2.2.3 Group stats, Reaction time. 6
2.3 Error rates 7
2.3.1 Bar Plot, error rates. 7
2.3.2 Group stats error rates. 8

3 ERP Data **9**
3.1 load data and generate mean amplitudes. 9
3.2 ERP Plots 11
3.2.1 ERPs on all electrodes 11
3.2.2 ERPs on midline electrodes 12
3.2.3 N2 Plot mean amplitudes CZ and FZ. 14
3.3 ERP Statistics 15
3.3.1 overview, descriptives on FZ 15
3.3.2 N2 Group statistics 16

1 Demograpics

Table A: All subjects, sex and age

Geschlecht	Alter.mean	Alter.min	Alter.max	Alter.length
female	23.2	22	26	10
male	24.5	21	30	11

2 Behavior data.

2.1 behavioral data, loading and exporting.

Table B: Behavior data, Header single data, find the full data in Supplement S2 File

names(VPNVERHALTEN_Export)
VPN
TARGET
DISTRACTOR
Error_Percent
RT_CorrectResponses
RT_ErrorResponses

Table C: Behavior data, Header group data, find the full data in Supplement S3 File

names(GROUPVERHALTEN_Export)
TARGET
DISTRACTOR
RT_mean
ErrorPercent_mean
RT_var
ErrorPercent_var
RT_sd
ErrorPercent_sd
RT.std.error
ErrorPercent.std.error

2.2 Reaction times

2.2.1 Descriptives, reaction time and errors.

Table D: Descriptives. RT in ms, Error in %

Target	Distractor	RT	RT_sd	Error%	Error%_sd
TRR	DC	688.8	56.0	8.4	5.4
TRR	DR	668.1	43.8	8.6	6.8
TRC	DC	705.4	46.8	8.2	5.3
TRC	DR	702.5	48.6	8.1	5.1

Table E: Main effect TARGET

Target	RT	RT_sd	Error%	Error%_sd
TRR	678.5	50.7	8.5	6.0
TRC	704.0	47.1	8.1	5.1

Table F: Main effect DISTRACTOR

Distractor	RT	RT_sd	Error%	Error%_sd
DC	697.1	51.7	8.3	5.3
DR	685.3	48.9	8.3	5.9

2.2.2 Bar graph reaction times.

- Bar graph reaction time is also provided in manuscript.

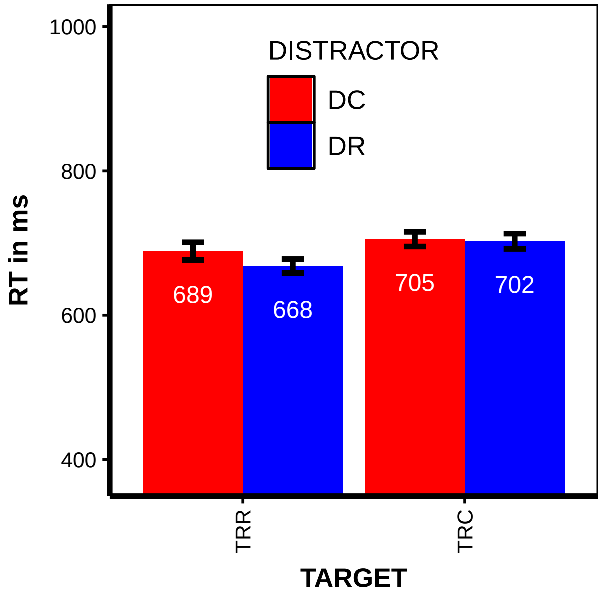

Figure A: Reaction time in ms

2.2.3 Group stats, Reaction time.

Table G: ANOVA Reaction times.

Effect	DFn	DFd	F	p	p<.05	ges	pes
(Intercept)	1	20	5203.4	0	*	1.0	1.0
TARGET	1	20	10.5	0	*	0.1	0.3
DISTRACTOR	1	20	8.1	0	*	0.0	0.3
TARGET:DISTRACTOR	1	20	7.2	0	*	0.0	0.3

Note: Column "ges" = generalized eta sqare, see Bakeman, 2005. pes = Partial Eta Squared

2.3 Error rates

2.3.1 Bar Plot, error rates.

- see table reaction times for descriptives.
- Error rates are only calculates for the sake of completeness, since the purpose of the practice trials was to minimize errors.
- See also Supplement S1 Fig

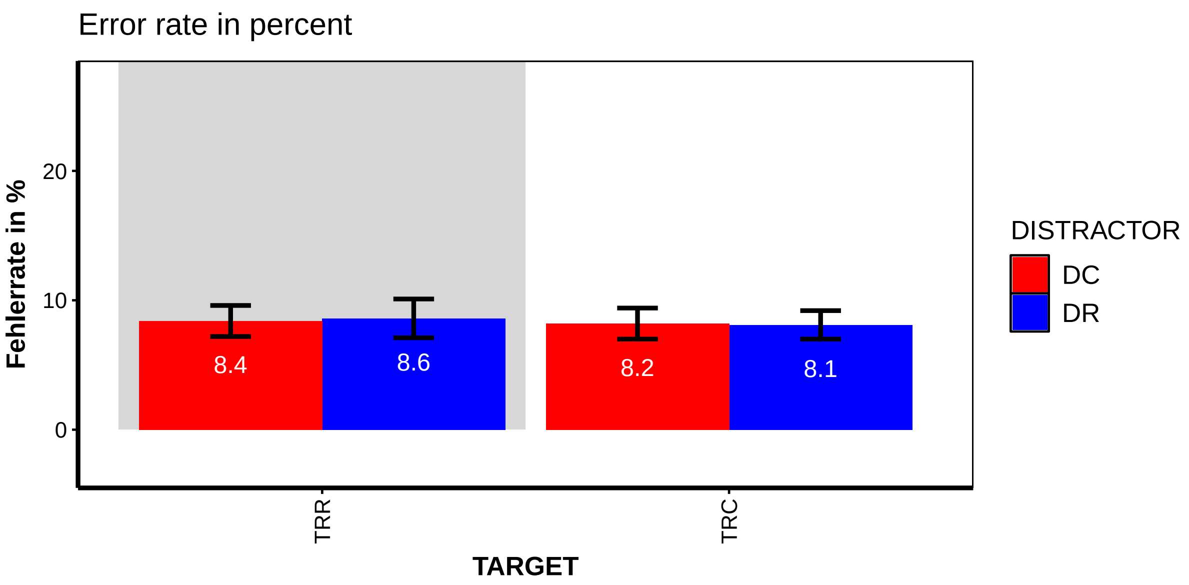

Figure B: Error rates in percent.

2.3.2 Group stats error rates.

Table H: Error percent ANOVA FULL

Effect	DFn	DFd	F	p	p<.05	ges	pes
(Intercept)	1	20	78.97	0.000	*	0.693	0.798
TARGET	1	20	0.11	0.747		0.001	0.005
DISTRACTOR	1	20	0.00	0.985		0.000	0.000
TARGET:DISTRACTOR	1	20	0.02	0.891		0.000	0.001

Note: Column "ges" = generalized eta sqare, see Bakeman, 2005. pes = Partial Eta Squared

3 ERP Data

3.1 load data and generate mean amplitudes.

- ERP data were exportet to txt from ERPlab and imported into R environment. Imported text files are provided. See Supplement “S4 File.txt“.

Table I: ERP data, Header single subjects, find the full data in Supplement S4 File

x
VPN
TARGET
DISTRACTOR
TIME
FP1
FP2
F3
F4
C3
C4
P3
P4
O1
O2
F7
F8
T7
T8
P7
P8
CZ
FZ
PZ
FC1
FC2
CP1
CP2
PO3
PO4
FC5
FC6
CP5
CP6
A1
A2

3.2 ERP Plots

3.2.1 ERPs on all electrodes

- Figure is also provided in supplement S2 Fig

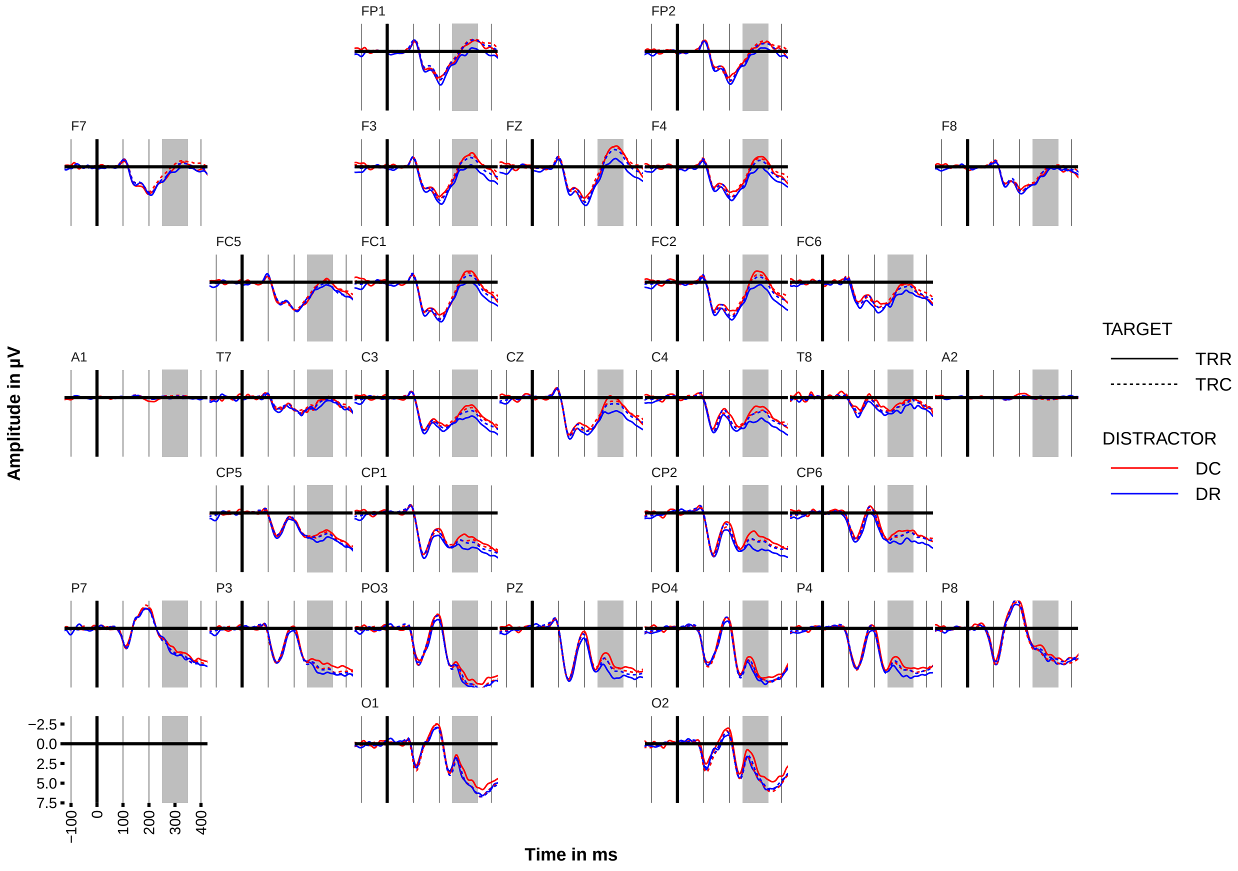

Figure C: ERPs on all electrodes. Grey box indicates time window for statistical analysis. See also S2 Fig for larger plot

3.2.2 ERPs on midline electrodes

- Figure is also provided in manuscript.

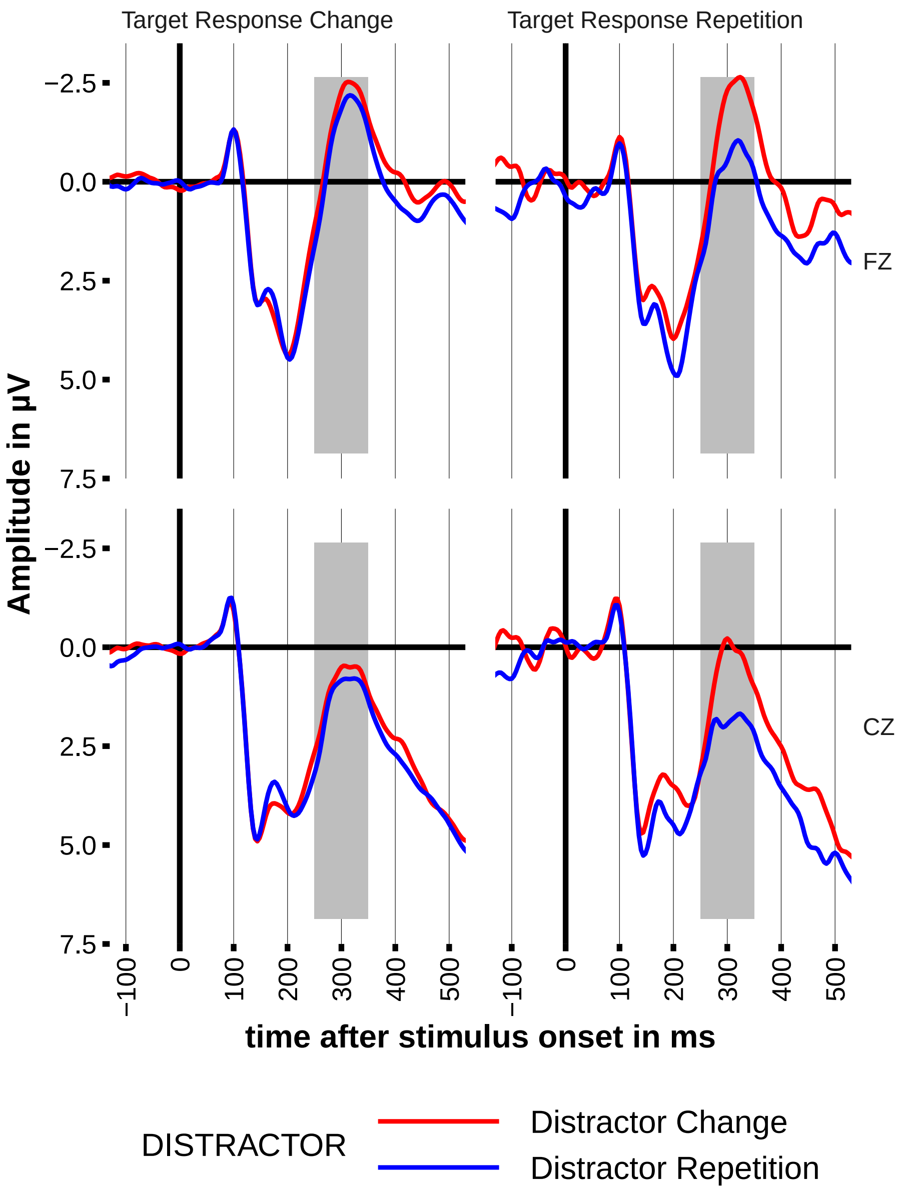

Figure D: ERPs on electrodes FZ and CZ, same figure as in manuscript. Grey box indicates time window for statistical analysis.

- See Supplement “S5 File.txt“ for mean values 250-350 ms for all Subjects.

Table J: ERP mean amplitudes 250-350 ms, Header single subjects, find the full data in Supplement S5 File

x
VPN
TARGET
DISTRACTOR
ELECTRODE
N2_mean250_350

Table K: Amplitudes, mean 250-350 ms, FZ & CZ, Descriptives

Measure	Target	Distr	Elec	mean	var
N2_mean250_350	TRR	DC	FZ	-1.43	13.34
N2_mean250_350	TRR	DC	CZ	0.72	14.63
N2_mean250_350	TRR	DR	FZ	-0.13	12.78
N2_mean250_350	TRR	DR	CZ	2.05	13.82
N2_mean250_350	TRC	DC	FZ	-1.51	14.15
N2_mean250_350	TRC	DC	CZ	1.00	13.52
N2_mean250_350	TRC	DR	FZ	-1.13	14.00
N2_mean250_350	TRC	DR	CZ	1.30	13.71

3.2.3 N2 Plot mean amplitudes CZ and FZ.

- see “S3 Fig.pdf“ for bar plot mean amplitude 250-350 ms on midline electrodes.

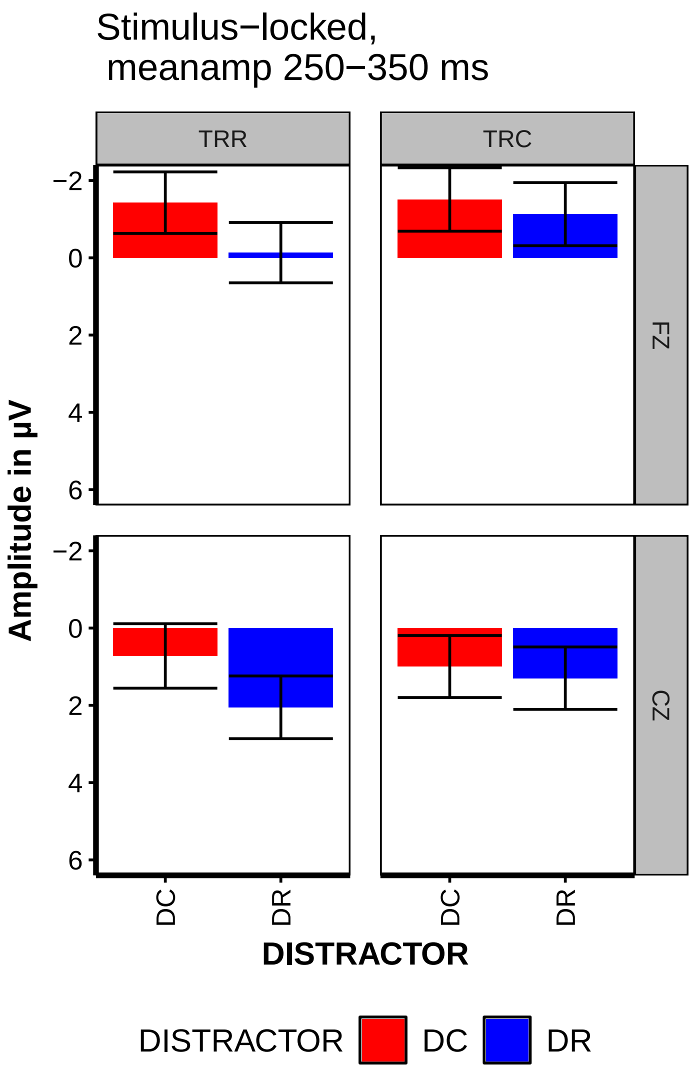

Figure E: mean amplitudes on electrodes FZ and CZ. See also Supplement S3 Fig, Error Bars are SE

3.3 ERP Statistics

3.3.1 overview, descriptives on FZ

- mean values for main effects and interaction TARGET x DISTRACTOR

Table L: N2 main effect TARGET on FZ

Target	mean	var	SD	stdE
TRR	-0.8	13.2	3.6	0.6
TRC	-1.3	13.8	3.7	0.6

Table M: N2 Main effect DISTRACTOR on FZ

Distr	mean	var	SD	stdE
DC	-1.5	13.4	3.7	0.6
DR	-0.6	13.3	3.6	0.6

Table N: N2 interaction TARGET and DISTRACTOR on FZ

Target	Distr	mean	var	SD	stdE
TRR	DC	-1.4	13.3	3.7	0.8
TRR	DR	-0.1	12.8	3.6	0.8
TRC	DC	-1.5	14.1	3.8	0.8
TRC	DR	-1.1	14.0	3.7	0.8

3.3.2 N2 Group statistics

Table O: Anova on ERP mean amplitudes.

Effect	DFn	DFd	F	p	p<.05	ges	pes
(Intercept)	1	20	0.02	0.886		0.001	0.001
TARGET	1	20	2.49	0.131		0.003	0.111
DISTRACTOR	1	20	13.80	0.001	*	0.013	0.408
ELECTRODE	1	20	24.30	0.000	*	0.093	0.549
TARGET:DISTRACTOR	1	20	4.77	0.041	*	0.004	0.193
TARGET:ELECTRODE	1	20	3.50	0.076		0.000	0.149
DISTRACTOR:ELECTRODE	1	20	0.02	0.902		0.000	0.001
TARGET:DISTRACTOR:ELECTRODE	1	20	0.12	0.731		0.000	0.006

Table P: Interaction TxDXE n.s. in full anova, therefore post hoc comparisons for two way interaction, TARGET x DISTRACTOR

TARGET1	DISTRACTOR1	sep	TARGET2	DISTRACTOR2	md	se	df	t	pnone	pscheffe	pbonferroni
TRR	DC	-	TRR	DR	-1.31	0.31	40.00	-4.17	0.00	0.00	0.00
TRR	DC	-	TRC	DC	-0.10	0.33	39.56	-0.29	0.77	0.99	1.00
TRR	DC	-	TRC	DR	-0.44	0.33	39.56	-1.31	0.20	0.64	1.00
TRR	DR	-	TRC	DC	1.22	0.33	39.56	3.66	0.00	0.01	0.00
TRR	DR	-	TRC	DR	0.88	0.33	39.56	2.63	0.01	0.09	0.07
TRC	DC	-	TRC	DR	-0.34	0.31	40.00	-1.08	0.29	0.76	1.00
