# Supplementary figures and images for: Useful distracting information: ERP correlates of distractors in stimulus-response-episodes
Source: PLoS One. 2018 Nov 1;13(11):e0206468. doi: 10.1371/journal.pone.0206468 (PMC6211706; doi:10.1371/journal.pone.0206468)

Error rate in percent

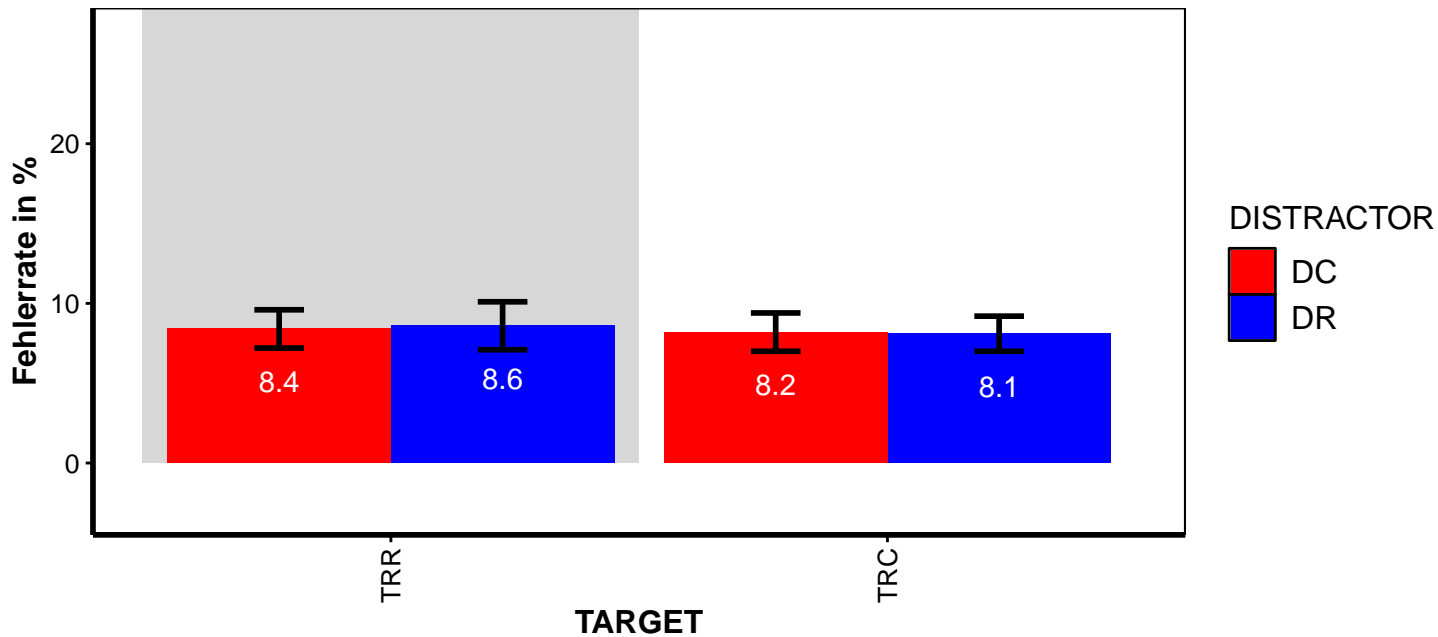

Supplement: S1 Fig — (PDF) [file pone.0206468.s006.pdf]

Amplitude in  $\mu\text{V}$

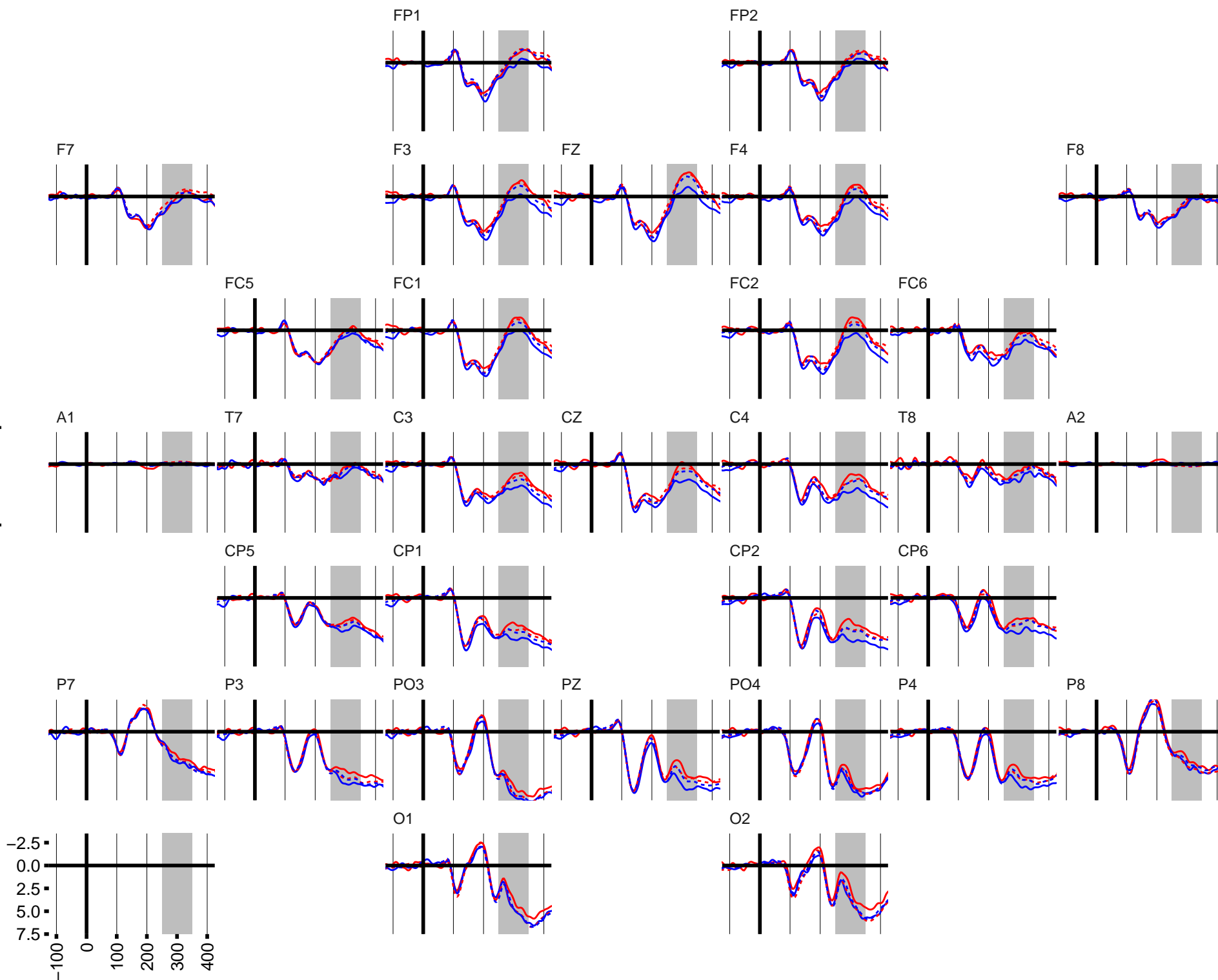

TARGET

—— TRR  
- - - - TRC

DISTRACTOR

— DC  
— DR

Time in ms

Supplement: S2 Fig — ERPs all electrodes by TARGET and DISTRACTOR. (PDF) [file pone.0206468.s007.pdf]

Stimulus-locked,  
meanamp 250–350 ms

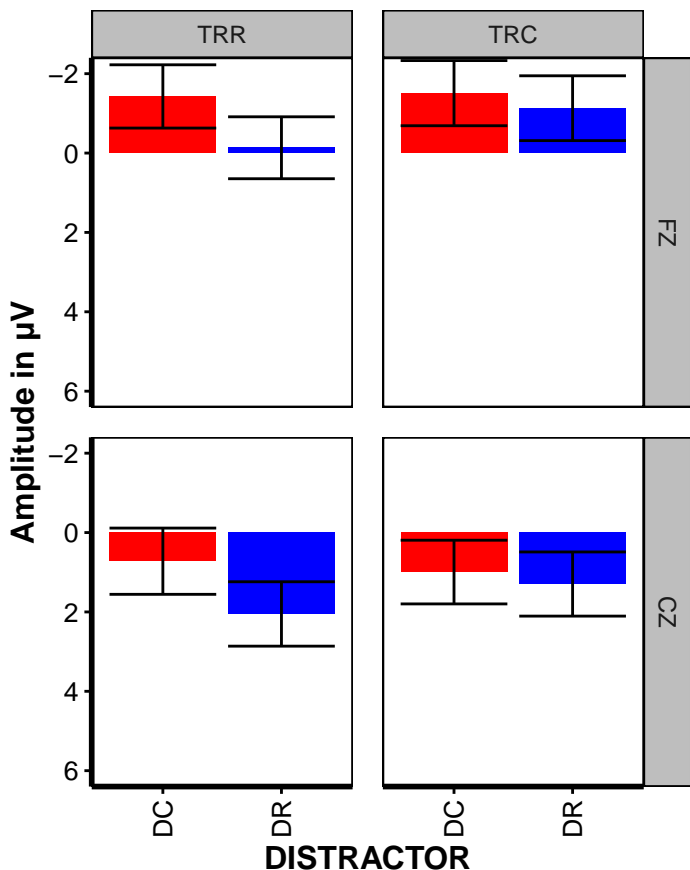

DISTRACTOR ■ DC ■ DR

Supplement: S3 Fig — N2 mean amplitudes on midline electrodes FZ and CZ. (PDF) [file pone.0206468.s008.pdf]
